# Acute hepatitis in three patients with systemic juvenile idiopathic arthritis taking interleukin-1 receptor antagonist

**DOI:** 10.1186/1546-0096-7-21

**Published:** 2009-12-22

**Authors:** Scott Canna, Jennifer Frankovich, Gloria Higgins, Michael R Narkewicz, S Russell Nash, J Roger Hollister, Jennifer B Soep, Leonard L Dragone

**Affiliations:** 1 Division of Rheumatology, The Children's Hospital, 13123 E 16th Ave, Aurora, CO 80045, USA; 2 Division of Rheumatology, Lucille Packard Children's Hospital, 725 Welch Rd, Palo Alto, CA 94304, USA; 3 Nationwide Children's Hospital, 700 Children's Drive, Columbus, OH 43205, USA; 4 Colorado GI Pathology, 7346 S. Alton Way, Suite 10-E, Centennial, CO 80112, USA; 5 Department of Pediatrics, National Jewish Health, 1400 Jackson St, Denver, CO 80206, USA

## Abstract

**Purpose:**

We investigated the etiology of acute hepatitis in three children with systemic Juvenile Idiopathic Arthritis (sJIA) taking Interleukin-1 receptor antagonist (IL1RA).

**Methods:**

Laboratory and clinical data for three children with sJIA diagnosed at ages 13 months to 8 years who developed acute hepatitis during treatment with IL1RA were reviewed for evidence of sJIA flare, infection, macrophage activation syndrome (MAS), malignancy, and drug reaction.

**Results:**

In all patients, hepatitis persisted despite cessation of known hepatotoxic drugs and in absence of known infectious triggers, until discontinuation of IL1RA. Liver biopsies had mixed inflammatory infiltrates with associated hepatocellular injury suggestive of an exogenous trigger. At the time of hepatitis, laboratory data and liver biopsies were not characteristic of MAS. In two patients, transaminitis resolved within one week of discontinuing IL1RA, the third improved dramatically in one month.

**Conclusions:**

Although sJIA symptoms improved significantly on IL1RA, it appeared that IL1RA contributed to the development of acute hepatitis. Hepatitis possibly occurred as a result of an altered immune response to a typical childhood infection while on IL1RA. Alternatively, hepatitis could have represented an atypical presentation of MAS in patients with sJIA taking IL1RA. Further investigation is warranted to determine how anti-IL1 therapies alter immune responsiveness to exogenous triggers in patients with immune dysfunction such as sJIA. Our patients suggest that close monitoring for hepatic and other toxicities is indicated when treating with IL1RA.

## Background

sJIA is a systemic autoinflammatory disorder of unknown etiology frequently characterized by quotidian fever, rash, generalized lymphadenopathy, hepatosplenomegaly, pericarditis and arthritis. Transaminitis, defined as elevation of hepatic enzymes (specifically alanine aminotransferase (ALT) and aspartate aminotransferase (AST)) in the absence of other clinical or laboratory evidence of hepatic injury, can be seen in patients with sJIA either in disease flares or due to hepatotoxic therapies like methotrexate [1,2]. The mechanism by which patients develop sJIA is unknown, but it is felt to involve the interaction of infectious or inflammatory triggers with a genetic predisposition toward an enhanced proinflammatory response. Cytokines, particularly Interleukin-1 (IL-1) and Interleukin-6 (IL-6) have been implicated as important in disease pathogenesis and maintenance of inflammation [1,2]. MAS is a complication of various rheumatologic and infectious diseases, and is often associated with sJIA. It is clinically characterized by cytopenias, disseminated intravascular coagulation, hemodynamic instability and liver and neurological involvement [3,4]. Hemophagocytosis by activated macrophages is considered by most authors to be the most specific histopathological feature of MAS [3,4]. Hemophagocytosis is generally accompanied by a lymphohistiocytic infiltrate including a predominance of CD8+ lymphocytes [5] although some reports in adults have additionally identified biliary injury and vascular microthrombi as frequent findings [6]. Mortality due to MAS has been estimated at 8-22% [3,7].

Non-steroidal anti-inflammatory drugs (NSAIDs) and systemic corticosteroids have historically been the mainstays of treatment for sJIA [1,2]. There is evidence that methotrexate, cyclosporine, and etanercept are less effective in sJIA than in other forms of juvenile arthritis, and some disease-modifying drugs may be associated with development of MAS [1,2]. IL-1 receptor antagonist (IL1RA) (anakinra) and anti-IL-6 (MRA/tocilizumab) have shown clinical efficacy in small open-label studies in sJIA and adult Still's Disease [8-11]. MAS, which leads to the greatest morbidity and mortality in patients with sJIA, is generally treated with supportive care, high dose intravenous corticosteroids and other immunosuppressive agents. MAS may also be treated with etoposide or cyclosporine in a similar fashion to primary or familial hemophagocytic lymphohistiocytosis (HLH), a genetic disorder related to alterations in cytotoxic granular release that can be triggered by infections and neoplasms [12]. MAS is a form of secondary HLH. There is a single case report of successful treatment of MAS with IL1RA in a patient with sJIA [13].

As many as 50-100% of patients with sJIA respond to IL1RA [8,10,11]. Despite these promising case series, the safety of IL1RA in large numbers of patients with sJIA has not been established. Furthermore, the effects of IL-1 blockade on the inflammatory response of patients with sJIA have not been formally studied. One case report suggests IL1RA as a trigger for MAS in an adolescent with sJIA [14]. In this case series we sought to investigate the extent to which a significant adverse event, acute hepatitis, was related to treatment with IL1RA in three patients with sJIA.

## Patients and Methods

Three patients with sJIA, all treated at different tertiary care pediatric centers, developed acute hepatitis while taking IL1RA. All laboratory and clinical data were collected and reviewed retrospectively (Table 1). All three patients had a presentation of severe disease with significant arthritis and persistently elevated inflammatory markers, but differed in age and ethnicity. All liver biopsies were reviewed by a single hepatopathologist (SRN). The Colorado Multiple Institutional Review Board approved this series.

## Results

**Figure 1:**
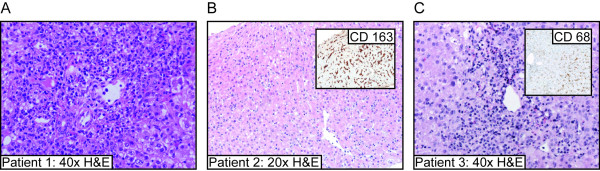
**Liver Biopsy results for three patients with SJIA and acute hepatitis**. (A) Patient 1 hematoxylin and eosin (H&E) staining of liver biopsy. (B) Patient 2 H&E staining of liver biopsy with inset picture of the liver biopsy stained with anti-CD163 antibodies (Kupffer cell marker) showing a moderate increase in sinusoidal macrophages. (C) Patient 3 H&E staining of liver biopsy with inset picture of the liver biopsy stained with anti-CD68 antibodies (macrophage marker) showing a mild increase in sinusoidal macrophages.

**Figure 2:**
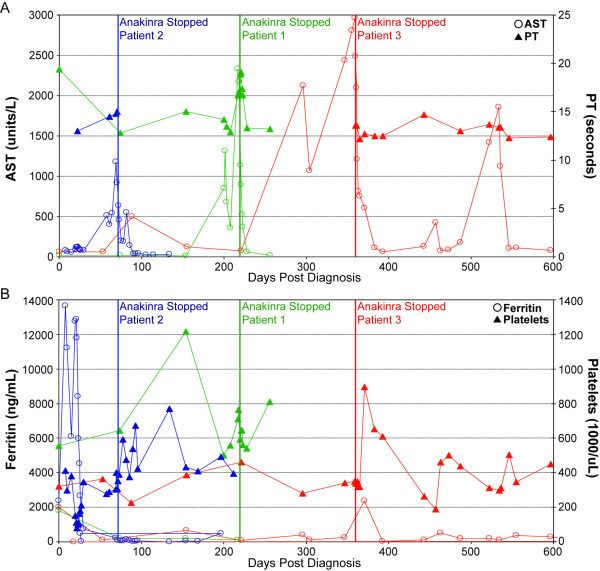
**Summary of lab findings from three patients with sJIA and hepatitis**. Green = Pt. 1, Blue = Pt. 2, Red = Pt. 3. (**A**) AST (open circles) and PT (filled triangles) (**B**) Ferritin (open circles) and Platelets (filled triangles).

After a thorough evaluation by a pediatric rheumatologist led to the diagnosis of sJIA, all three patients were treated with IL1RA in the first few months after diagnosis due to severe or refractory disease (Table 1). Patients 2 and 3 were treated with IL1RA without prior methotrexate or TNFα-inhibiting therapies, and both patients had received treatment with high-dose IV corticosteroids in the month preceding hepatitis. After development of abdominal pain in all patients and jaundice in 2 patients, acute hepatitis was diagnosed between 44 and 250 days after initiation of IL1RA treatment. A thorough evaluation of each patient's hepatitis ruled out common infectious and autoimmune causes; although antecedent flu-like symptoms were present in patient two, and fever/diarrhea in patient three. Patient one had neither viral symptoms nor serologic evidence of an infectious process or drug exposure; although she had been treated with methotrexate until two weeks prior to the development of hepatitis. Liver biopsies showed a mixed inflammatory infiltrate without hemophagocytosis or microthrombi, suggesting hepatocellular injury due to an exogenous trigger. Two patients had significant hyperbilirubinemia with increased serum direct bilirubin (data not shown), without histological cholestasis on liver biopsy. Elevation of cholestatic enzymes was less impressive than transaminitis, and patient two had normal measurements. Cessation of IL1RA resulted in rapid improvement of liver enzymes, but two patients experienced a sJIA flare, requiring pulse corticosteroid therapy. Clinical suspicion for MAS was low, and bone marrow biopsies were not performed. Liver biopsies showed a nonspecific pattern of mixed inflammation without identifiable hemophagocytosis, vascular microthrombi, or cholestatic biliary injury. Staining for macrophage markers (CD68 and CD163) showed a mild to moderate increase in the numbers of Kupffer cells, which may represent a nonspecific reaction to hepatocyte injury (Figure 1). Patient 1 also showed evidence of liver synthetic dysfunction (prolongation of her PT) that improved rapidly after cessation of IL1RA (Figure 2). Patient one had no LFT elevations prior to treatment with IL1RA, while patient 2 had mild transaminitis with MAS just prior to initiation of IL1RA, and patient 3 had multiple bouts of transaminitis with flu-like illnesses both before and after her acute hepatitis (Figure 2A). Thrombocytopenia and elevated ferritin, known markers of MAS, were absent during the time period of acute hepatitis in all patients (Figure 2B). Patient 1 and Patient 2 were restarted on IL1RA for refractory disease, and have had no further liver problems.

## Discussion

This case series is illustrative of three children with sJIA who developed acute hepatitis while being treated with IL1RA. Clinical and laboratory data, as well as liver biopsy, were not characteristic of MAS. Biopsies instead suggested hepatocellular injury from an exogenous cause, be it infectious or chemical (Figure 1). The presence of eosinophils in two of the three liver biopsies may also support an exogenous trigger contributing to the hepatitis. A viral trigger may have contributed to development of hepatitis in two patients, although serologies for viruses known to induce hepatitis were negative (Table 1). We are then left with a serious adverse event coincident with IL1RA treatment and reversed upon discontinuation of the drug. This suggests that IL-1 blockade contributed to the development or propagation of these patients' liver injury.

The clinical history of these patients underscores our lack of understanding of the influence of IL-1 blockade on the natural history of sJIA. Hepatitis could occur due to four potentially overlapping mechanisms. First, pure drug toxicity from anakinra appears possible, but unlikely given the absence of prior reports of hepatitis with anakinra therapy, the variable time to onset of hepatitis, and the tolerance of restarting IL1RA in two patients. Second, induction of autoimmune hepatitis (AIH) by the use of a biologic agent is possible, but also appears unlikely in the absence of specific autoantibodies or histopathological features associated with AIH. Third, immune dysregulation induced by IL1RA in children with sJIA could lead to hepatitis upon exposure to a normally benign infectious or environmental trigger. Fourth, IL1RA could change the clinical manifestations of MAS flare in some patients with sJIA leading only to acute hepatitis instead of the full MAS phenotype.

In vitro evidence suggests that inflammatory cytokines have pleiotropic effects on hepatocytes that can influence the development of hepatitis. Tumor necrosis factor receptor 1 (TNFR1), which can be expressed on hepatocytes, can signal through the TNFR-associated death domain (TRADD) to induce caspase activation and apoptotic cell death of hepatocytes [15]. Signaling through TNFR1 can also lead to the formation of reactive oxygen species, sustained c-Jun N-terminal kinase (JNK) activation and apoptotic cell death via a caspase-independent mechanism [16]. In contrast, signaling through TNFα and IL-6 receptors on hepatocytes can elicit signals that promote liver regeneration [17,18]. These apparently contradictory findings underscore our poor understanding of the roles of cytokines in liver injury. IL1RA may change cytokine production and response in patients with sJIA, and in a subset of patients this could lead to direct effects on hepatocyte apoptosis and the development of hepatitis.

This case series is important in that it is the first account of acute hepatitis in children with sJIA treated with IL1RA. Hepatitis appears to have occurred outside the context of a sJIA flare or the development of MAS. The short half-life of IL1RA may have been advantageous, since discontinuation led to the rapid improvement of hepatitis. Newer IL-1 blocking agents with longer half-lives could have a more prolonged effect in susceptible patients with sJIA. Data in patients with sJIA and Still's Disease are emerging that support the efficacy and safety of IL-1 blockade, and use of these treatments will likely expand [2,8,10,11]. Attention should be paid to possible hepatic side effects in clinical trials and post-marketing safety studies. Frequent LFT monitoring as well as the capacity for rapid reversal of IL-1 blockade may be important in preventing liver damage in some patients. This case series highlights the importance of more prospective studies such as the one recently published by Gattorno *et al *to determine the influence of IL-1 blockade on the cytokine networks in patients with sJIA [19].

## Consent

Written informed consent was obtained from the parents of these patients for publication of this case report and any accompanying images. A copy of the written consent is available for review by the Editor-in-Chief of this journal.

## Competing interests

The authors declare that they have no competing interests.

## Authors' contributions

SC collected and organized data, and drafted the manuscript. JF & GH assisted in data collection and analysis, and contributed to the manuscript. MRN provided help with analysis of hepatologic data and contributed to the manuscript. SRN independently reviewed the biopsies and contributed to the manuscript. JRH & JBS provided help with analysis of rheumatologic/MAS data and contributed to the manuscript. LLD oversaw the project, critically reviewed the manuscript and provided rheumatologic and immunologic analysis of the data.
